# Industry 4.0 at the Service of Public Health Against the COVID-19 Pandemic

**DOI:** 10.1017/dmp.2021.286

**Published:** 2021-09-09

**Authors:** Giandomenico Nollo, Francesco Pilati, Riccardo Tronconi, Marta Rigoni

**Affiliations:** 1 Department of Industrial Engineering, University of Trento, Italy; 2 Department of Biomedical, Surgical and Dental Sciences, University of Milan, Italy

**Keywords:** public health mass intervention, digital twin model, asymptomatic sars-cov-2 detection, covid-19, mass screening, mass vaccination

During November 2020, the Alpine Italian province of Bolzano planned a mass SARS-CoV-2 screening on its population of 500000 in order to slow down infections and alleviate hospitals’ pressure. The province aimed to screen from Friday to Sunday, using the Rapid Antigen Test (RAT) on 350000 people, which was about 75% of the population over the age of 5. It called on citizens to participate on a voluntary basis after collecting their signed informed consent.

Mass SARS-CoV-2/COVID-19 testing was seen as a valuable tool for identifying and isolating asymptomatic carriers of the new coronavirus. These carriers are considered mainly responsible for the infection spread in the current resurgence of the pandemic. RATs received an emergency use authorization from European and US Agencies, and could be the key to boosting testing capacity because of their lower economic, time, and labor costs compared to the molecular tests.^1^ Despite their capacity to meet the most urgent needs, RATs suffered from a high number of false negative results, mostly in asymptomatic citizens, being affected by low tests’ sensitivity (on average 56% cross-study).^2^

## Tests

At the end of the 3-day campaign employing 740 healthcare professionals and 560 administrative operators, 352176 tests through nasopharyngeal swab [almost equally distributed between Abbott-Panbio^TM^ COVID-19 Ag (Abbott Laboratories, Illinois, U.S.) and SD Biosensor-STANDARD^TM^ Q COVID-19 Ag (SD Biosensor, Gyeonggi-do, Korea)] were administered, and 3380 positive asymptomatic subjects were identified. After a first ramp-up of the procedures, the testing campaign was efficiently organized with limited queues and a remarkable satisfaction of the general population.

Alongside appropriateness and performance of RATs and of healthcare interventions, the logistical aspect of such a large-scale intervention of the population is also of great interest and has multiple public health implications concerning potential logistic inefficiency such as lack of materials, transparency, access times, and queues. Limiting the following risks during the testing campaign execution was of major importance:

- contagion between citizens and operators
- public disorder arising from inefficiency of the service
- citizen disaffection with respect to public health policies
- operators overload and burnout

## Model Development and Effectiveness

In order to support the mass test deployment, a tailored digital twin model was developed. This technology comes from Industry 4.0 and consists of a digitalization of the production/service system while providing a highly integrated value chain.^3^ Industry 4.0 quantitative models are widely used in manufacturing but their usefulness has not yet been demonstrated in the healthcare context. The potential of this approach is enormous and it was elicited in this mass public health intervention by designing, with the highest accuracy, 194 test sites and their key features as types and numbers of personnel to be involved (nurses, administrative staff, etc.), consumables (gloves, protective suits, etc.), necessary spaces (in squared meter for each testing phase), intervention execution time (average patient time inside clinic), and queue length to guarantee adherence to the physical distance requirements.^4^

The Italian experience could represent a turning point in the pandemic contrast policies as it addressed relevant elements for public health interventions planning. Considering the huge logistical challenge to be faced with the upcoming massive worldwide vaccination in the next months,^5^ an Industry 4.0-based approach could be successfully leveraged to design the COVID-19 vaccination campaign by determining human, supply, and space resources; and minimizing time, as well as citizens’, and operators’ inconvenience, while providing the best safety guarantees.
